# Case report: Esophageal bulge with white patch: endoscopic removal of leiomyoma and high-grade intraepithelial neoplasia

**DOI:** 10.3389/fonc.2024.1515288

**Published:** 2024-12-06

**Authors:** Lili Pan, Chong Zhang, Ran Ma, Lijuan Fan

**Affiliations:** ^1^ Department of Gastroenterology, Jining First People’s Hospital, Jining, China; ^2^ Department of Pathology, Jining First People’s Hospital, Jining, China

**Keywords:** esophageal leiomyoma, esophageal bulge, high-grade intraepithelial neoplasia, leukoesophageal plaques, endoscopic resection

## Abstract

Esophageal leiomyoma is the most common benign intramural tumor of the esophagus. Despite being the most common benign tumor in its category, esophageal leiomyomas constitute only 1.2% of all esophageal tumors. While esophageal leiomyoma itself is uncommon, the occurrence of multiple esophageal leiomyomas is even rarer, and the coexistence of high-grade squamous intraepithelial neoplasia (HGIN), involving both epithelial and mesenchymal tissues, is exceedingly rare. This case report describes a patient with multiple esophageal leiomyomas and localized HGIN, diagnosed using endoscopic ultrasonography, which identified a submucosal lesion within the muscularis mucosae. The lesion was successfully treated using endoscopic high-frequency electrocoagulation resection. This minimally invasive approach proved to be precise, safe, and effective, offering therapeutic outcomes comparable to those of traditional surgical resection, as confirmed by postoperative pathological analysis. As a primary goal, the abstract should render the general significance and conceptual advance of the work clearly accessible to a broad readership. References should not be cited in the abstract. Leave the Abstract empty if your article does not require one – please see the “Article types” on every Frontiers journal page for full details.

## 1 Introduction

Esophageal leiomyoma is a rare benign tumor that arises from the smooth muscle cells of the esophageal wall (1, 2). The incidence of benign esophageal tumors ranges from 0.005% to 7.9%, with leiomyomas accounting for 70-80% of these cases (3). It is often asymptomatic and discovered incidentally during imaging studies. Most patients with esophageal leiomyomas do not exhibit significant clinical symptoms; however, larger tumors (>5 cm) may cause dysphagia or chest pain (4, 5). These tumors are most commonly observed in individuals between 20 and 50 years of age, with a slight male predominance. While the exact etiology remains unclear, they are believed to result from localized smooth muscle hyperplasia or genetic mutations (6).

Although surgical resection has traditionally been the standard treatment for symptomatic esophageal leiomyomas, recent advances in endoscopic techniques, such as endoscopic ultrasonography (EUS) and high-resolution magnification endoscopy, have provided safer, minimally invasive alternatives for both diagnosis and treatment (6–8). However, the management of esophageal leiomyomas with concomitant early superficial lesions presents a challenge.

This case report discusses a rare instance of multiple esophageal leiomyomas in an elderly patient, complicated by localized high-grade squamous intraepithelial neoplasia (HGIN). The lesion was diagnosed through the use of EUS, chest imaging, and endoscopic pathology. High-frequency electrocoagulation resection was successfully performed via endoscopy to treat the lesion. The aim of this report is to delve into the role of advanced endoscopic techniques in the diagnosis and treatment of esophageal leiomyomas, especially in cases involving concurrent superficial lesions, and to review current management strategies for this condition.

## 2 Case report

**Figure 1:**
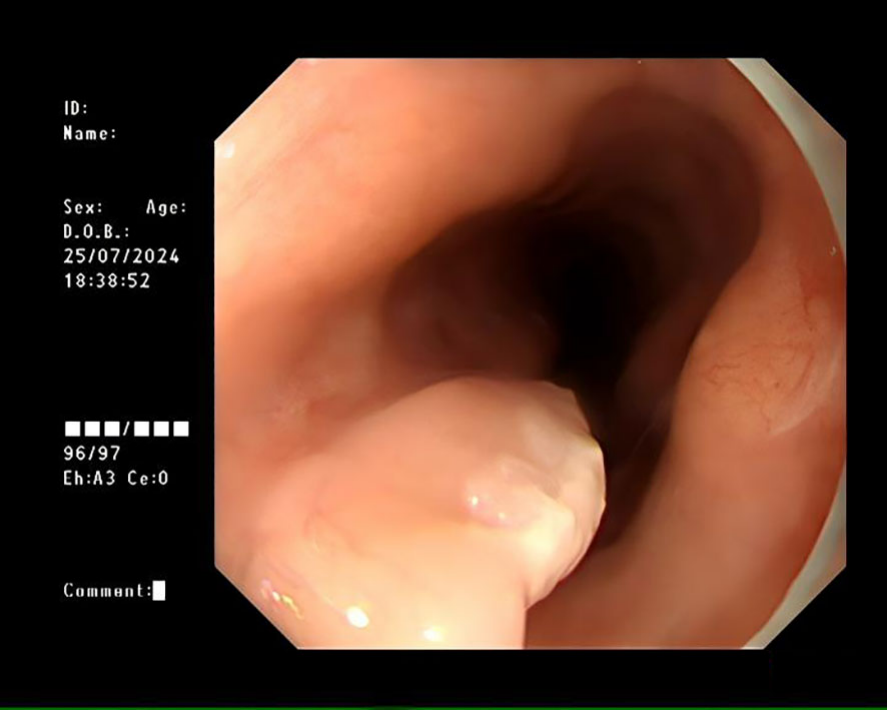
Submucosal protrusion in the esophagus with a smooth surface, partially covered by white patches, as observed under white light endoscopy.

**Figure 2:**
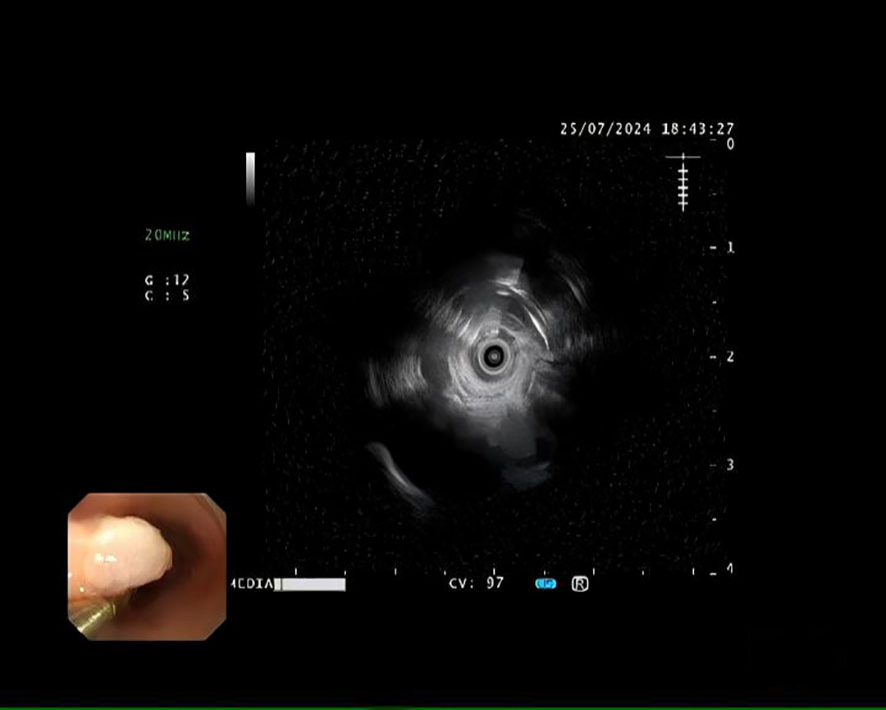
Hypoechoic tumor measuring approximately 12 mm in diameter, confined to the muscularis mucosae, with a smooth contour and well-defined borders, as seen on endoscopic ultrasonography.

A 72-year-old female was admitted for persistent abdominal pain lasting 4 months. She had no prior history of tumors or related diseases. On admission, chest and abdominal CT scans revealed no tumor lesions. There was no obvious thickening of the esophagus, and no enlarged lymph nodes around the esophagus were observed. Endoscopy showed a smooth-surfaced lesion on the anterior wall of the esophagus, approximately 1.2×0.6 cm in size, located 17 cm from the incisors. Another submucosal protrusion, approximately 1.2×0.6 cm in size, was found on the anterior esophageal wall, 20 cm from the incisors, partially covered by a white plaque (**Figure 1**). EUS of both lesions revealed clear and intact layers 1, 3, and 4, with a uniform hypoechoic area observed in the second layer, suggesting that the lesions originated from the mucosal muscularis (**Figure 2**). Both lesions were excised using high-frequency electrocoagulation, with no bleeding at the resection margins. Four hemostatic clips were used to close the wound bed.

**Figure 3:**
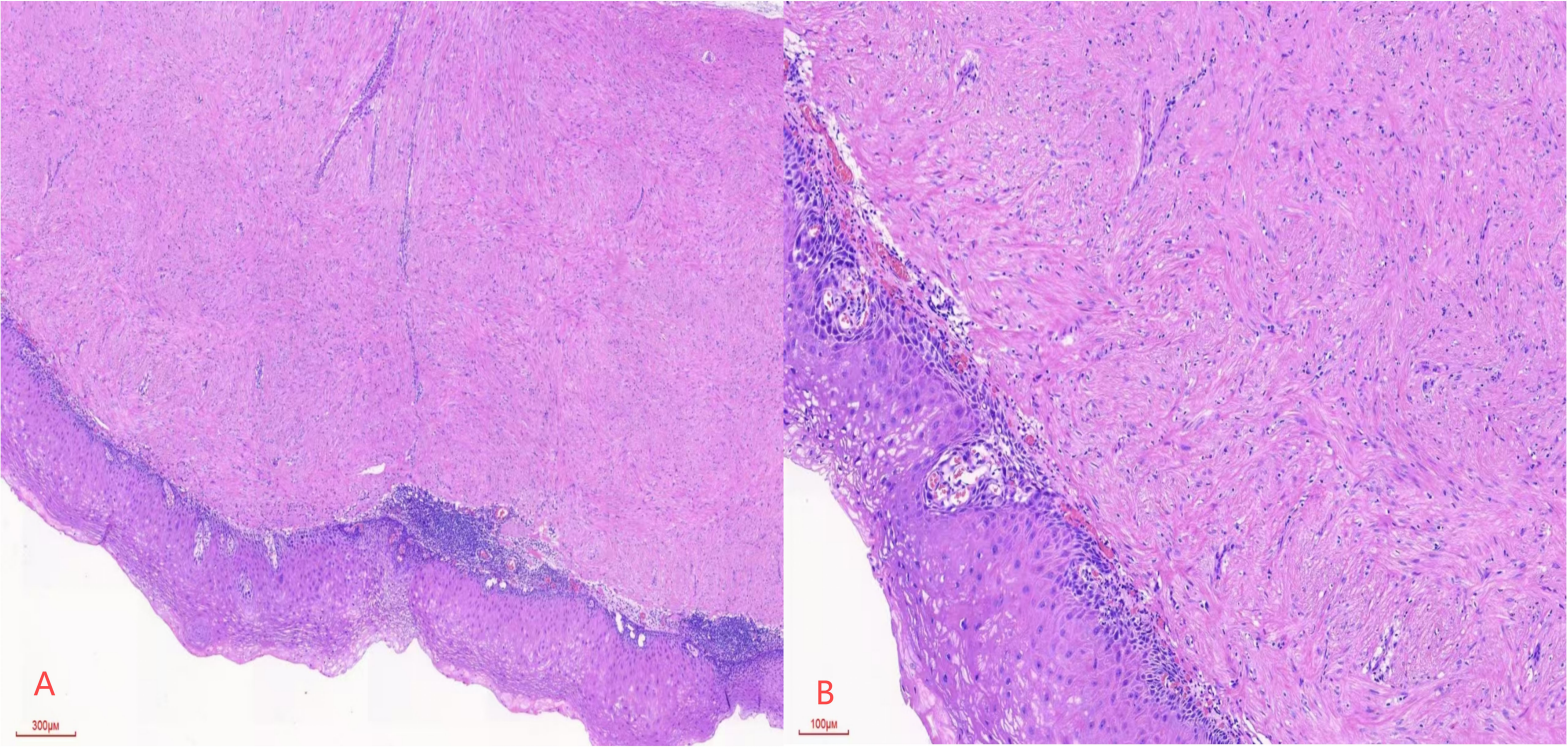
**(A)** Spindle cells are arranged in a fascicular pattern, with the surface covered by atypical squamous epithelium, at a magnification of HE×40. **(B)** Squamous epithelial cells exhibit enlarged, atypical, and hyperchromatic nuclei, with the lesion extending beyond half of the epithelial layer, at a magnification of HE×100.

Postoperative pathology revealed esophageal leiomyoma (17 cm from the incisors) and esophageal leiomyoma with high-grade squamous intraepithelial neoplasia (20 cm from the incisors). **Figure 3A** shows that after HE staining at ×40 magnification, spindle-shaped cells grow in bundles, with the surface covered by atypical squamous epithelium. **Figure 3B** shows that after HE staining at ×100 magnification, the squamous epithelial cell nuclei are enlarged, atypical, and deeply stained, with the lesion area involving more than half of the affected area. The diagnosis is high-grade squamous intraepithelial lesion (HGIN) of the esophagus.

Submucosal protrusions with white plaques in the esophagus represent a pathological condition that includes both smooth muscle tumors and squamous intraepithelial lesions. In pathological diagnosis, immunohistochemical markers such as Ki67, p53, SMA (smooth muscle actin), and Desmin play important roles in distinguishing smooth muscle tumors from squamous lesions. Ki-67 expression in tumor tissue can serve as a non-invasive biological marker for predicting tumor prognosis (9). In high-grade intraepithelial lesion areas, Ki67 expression is usually high and can assist in evaluating disease progression. p53 is a tumor suppressor protein, and studies have shown that using immunohistochemical detection of p53 mutations can diagnose high-grade atypical squamous epithelial hyperplasia in the esophagus (10). Mutations and overexpression of p53 may be associated with the malignant potential of smooth muscle tumors. SMA (smooth muscle antibody) (97.2%) and Desmin (94.5%) are positive in most smooth muscle tumor patients, serving as markers of smooth muscle cells and being helpful in confirming myogenic tumors (11). In squamous intraepithelial lesions, SMA and Desmin are usually negative. Ki67 and p53 help assess tumor proliferative activity and the risk of malignant transformation, while SMA and Desmin confirm the smooth muscle origin of the tumor. The expression patterns of these markers are useful for distinguishing smooth muscle tumors from squamous lesions. In pathological diagnosis, a comprehensive analysis of the expression of these markers along with other clinical and pathological features can provide more accurate diagnosis and treatment strategies for the patient.

In this case, immunohistochemical results showed localized high expression of Ki67, p53 mutation-positive staining, and positive expression of SMA and Desmin, which strongly supports the diagnosis of esophageal smooth muscle tumor with high-grade intraepithelial lesion. Combined with EUS examination, the lesion was located in the mucosal muscularis. Endoscopic high-frequency electrocoagulation was used to remove the esophageal lesion. Postoperative pathology remains the gold standard for diagnosis.

## 3 Conclusion

This case report presents a rare coexistence of esophageal leiomyoma and HGIN, accompanied by local white plaque on the surface of a submucosal protrusion. By combining computed tomography (CT), EUS, and EUS-guided biopsy, we were able to determine the depth of the lesion and the nature of the surface white plaques, providing guidance for our clinical treatment and offering a new perspective for our clinical diagnosis. Through high-frequency electrocautery endoscopic resection, we successfully treated the esophageal leiomyoma and removed the lesion with the associated white plaque. The results demonstrated that this treatment method is precise, minimally invasive, quick to recover, safe, and low-risk, achieving comparable outcomes to traditional surgical resection. In rare cases of concurrent esophageal leiomyoma and high-grade squamous intraepithelial neoplasia, particularly when white plaque suggests high-grade intraepithelial neoplasia, the combination of EUS, narrow-band imaging (NBI), and magnified endoscopy for evaluating lesion depth and plaque characteristics provides reliable clinical guidance, allowing the patient to achieve optimal treatment outcomes while avoiding radical esophageal resection.

## 4 Discussion

Rare Disease Coexistence: Esophageal leiomyoma is a rare benign tumor, typically characterized by the proliferation of smooth muscle cells within the esophageal wall, leading to localized thickening (5). Most esophageal leiomyomas are solitary lesions involving only smooth muscle cells (12). However, the simultaneous occurrence of esophageal leiomyoma and high-grade squamous intraepithelial neoplasia (HGIN) is exceedingly rare. It involves both abnormal proliferation of esophageal epithelial cells and changes in the mesenchymal cell components. High-grade squamous intraepithelial neoplasia is characterized by abnormal epithelial cell proliferation, accompanied by poor differentiation and increased nuclear atypia, and is generally considered a precancerous lesion that significantly increases the risk of developing esophageal squamous cell carcinoma (ESCC) (4, 13, 14). Therefore, early evaluation and intervention of epithelial changes are crucial to prevent malignant transformation. In addition, the proliferation of mesenchymal cells forms the main part of the tumor in esophageal leiomyoma (15). This proliferation may alter the normal structure and function of the esophagus, affecting its physiological state. Notably, In complex lesions involving esophageal leiomyoma and high-grade squamous intraepithelial neoplasia (HGIN), mesenchymal cells play crucial roles across various aspects of tumor biology, including the tumor microenvironment, immune regulation, and epithelial-mesenchymal interactions. These cells provide essential structural support within the tumor microenvironment, with smooth muscle cells in esophageal leiomyoma forming a framework that facilitates tumor growth and development. By regulating the extracellular matrix, mesenchymal cells promote tumor cell proliferation and migration, offering both physical space and biochemical support for tumor progression (1). Furthermore, mesenchymal cells actively engage in epithelial-mesenchymal interactions by secreting cytokines, chemokines, and growth factors, such as TGF-β and HGF. These factors encourage the proliferation, migration, and malignant transformation of epithelial cells, accelerating tumor progression through localized signaling networks, especially in the presence of HGIN (16, 17). Mesenchymal cells also contribute to immune regulation within the tumor microenvironment by secreting immune-suppressive factors like IL-10 and TGF-β, which help the tumor evade immune surveillance. This immune evasion mechanism enhances the tumor’s ability to develop undetected, thereby increasing its invasiveness (18). Additionally, mesenchymal cells promote fibrosis and tissue remodeling, which can worsen tumor progression. In esophageal leiomyoma, fibrosis is linked to the activation and proliferation of smooth muscle cells, which alter the tissue architecture and create a microenvironment conducive to continuous tumor growth. This fibrotic process, particularly in the context of coexisting HGIN, further increases the risk of tumor progression, as fibrosis often involves matrix hardening and immune escape (19, 20). Moreover, mesenchymal cells can drive epithelial-mesenchymal transition (EMT), a key process in tumor progression where epithelial cells lose polarity and cell junctions, acquiring a mesenchymal-like phenotype that enhances invasiveness. EMT activation in the context of HGIN may be influenced by signals from surrounding mesenchymal cells, promoting tumor cell migration, invasion, and metastasis (21, 22). Ultimately, mesenchymal cells, through their multifaceted roles in modulating the tumor microenvironment, immune response, fibrosis, and EMT, serve as a central driving force in the progression of esophageal leiomyoma and high-grade squamous intraepithelial neoplasia.

Innovative Perspective in Clinical Diagnosis: In this case, multiple esophageal leiomyomas was initially diagnosed by EUS, with the lesion presenting as a well-defined, smooth-edged mass, suggesting a benign tumor. The lesion was located in the mucosal muscularis and showed characteristic features of a typical esophageal leiomyoma: the tumor was sessile, with well-defined borders composed of smooth muscle bundles and a fibrous capsule. The lesion was small, multiple, eccentric, firm, and round in shape, with easy separability. The tumor was successfully removed by endoscopic high-frequency electrocautery, and histopathological examination confirmed the diagnosis. Research has shown that esophageal leiomyomas may have a protective role by preventing the spread of overlying squamous cell carcinoma and preventing deeper infiltration (12, 23). In this case report, the presence of high-grade squamous intraepithelial neoplasia in conjunction with the leiomyoma suggested that the lesion was at an early superficial stage. Although such cases are rare, esophageal submucosal tumors can sometimes be associated with squamous cell carcinoma, making it essential to accurately assess the presence and depth of squamous cell carcinoma. This requires further diagnostic investigations such as chest and abdominal CT scans to evaluate potential distant metastasis or abdominal lymph node involvement. Esophageal squamous cell carcinoma is the most common epithelial malignancy of the esophagus (24). For differential diagnosis of esophageal leiomyoma with leukoplakia, it is recommended to perform multiple endoscopic biopsies to obtain more accurate pathological information. Chest and abdominal CT scans are also crucial for assessing the size and extent of leukoplakia. NBI endoscopy enhances tissue contrast and scattering effects, helping to better identify lesions, especially when combined with magnifying endoscopy for histological analysis and lesion assessment, which has significant clinical value. Therefore, by combining CT, EUS and EUS-guided biopsy, we were able to accurately assess the depth and extent of the leukoplakia lesion, leading to a precise diagnosis. Further evaluation using narrow-band imaging (NBI) and magnification endoscopy provided a new perspective on the lesion depth, offering enhanced clinical diagnostic insight and ensuring both accuracy and comprehensiveness in the diagnosis.

Innovative Treatment Approach: This case utilized high-frequency electrocoagulation for endoscopic resection of the multiple esophageal leiomyomas, successfully removing the lesion accompanied by local leukoplakia. The results indicate that this treatment method is precise, minimally invasive, offers rapid recovery, is safe, and carries low risks, achieving results comparable to traditional surgical resection. In the rare case of coexisting multiple esophageal leiomyomas and HSIL, especially when leukoplakia suggests high-grade intraepithelial neoplasia, the complexity of diagnosis and treatment increases and should not be overlooked. The use of EUS, NBI, and magnification endoscopy to assess lesion depth and the nature of the leukoplakia provided reliable guidance for clinical decision-making, allowing the patient to achieve optimal treatment outcomes while avoiding the need for radical esophagectomy.

## Data Availability

The raw data supporting the conclusions of this article will be made available by the authors, without undue reservation.
